# Determinants of supply chain coordination of milk and dairy industries in Ethiopia: a case of Addis Ababa and its surroundings

**DOI:** 10.1186/s40064-015-1287-x

**Published:** 2015-09-16

**Authors:** Habtamu Regassa Lemma, Rajwinder Singh, Navjot Kaur

**Affiliations:** School of Management Studies, Punjabi University, Patiala, India; International Management Institute, Bhubaneswar, India

**Keywords:** Coordination, Supply chain management, Ethiopian dairy industries, Structural equation modeling

## Abstract

Coordination of different business activities among units becomes vital as organizations pay much attention to their core activities. Thus, their fruitfulness constantly relies on their capacity to coordinate their internal and external activities in the supply chain outside their own boundaries. Giving consideration to these obvious reasons, this paper aims to investigate the determinants of supply chain coordination of milk and dairy industries. The data were collected from 330 milk suppliers, processors, and retailers in the central part of Ethiopia. The structural equation modeling has been employed to develop the structural relationship between key constructs and measured variables. In total, 15 measured variables for coordination in the supply chain have been identified. These are further grouped into four factors namely, non-price coordination, price coordination, relationships and product development decision. It has been observed that the implementation of these factors could maximize the coordination linkage among supply chain members. Thus, dairy sectors should take the identified coordination factors into account in each of their business dealings.

**Electronic supplementary material:**

The online version of this article (doi:10.1186/s40064-015-1287-x) contains supplementary material, which is available to authorized users.

## Introduction

As it is clearly seen in the business environment nowadays, effective supply chain management seems to be considered as a crucial concern that has to be dealt with in a global business context (Haghighat 2008). In the local activities of traditional business, those involving in the supply chain have been doing such activities independently. But at present, it is not advised to perform business independently considering the ever growth of the competitive market (Xu and Beamon 2006). Consequently, more developed and well-organized supply chain coordination is ideal for consistent success and profitability of any business. The most convincing reason for such claim is that the ever increasing competition that is constantly influenced by business globalization, product diversity and technological advancement motivated independent firms to work in unity in a supply chain that allows them to gain mutual benefits (Thomas and Griffin 1996).

Since a supply chain consists of various organizations, it can satisfy customers’ needs, only when the whole of its partners becomes integrated and coordinated (Haghighat 2008). In this way, supply chain drivers ought to jointly create value and improve supply chain performance effectively and efficiently (Lewis and Talalayevsky 2004). Even if the objectives and interests of different supply chain members are varied, the coordination among them becomes undeniably crucial to determine the supply chain performance as a whole (Ning et al. 2008).

In trying to elaborate the attributes of coordination, we can say that coordination in a supply chain involves putting the existing interdependencies in order (Li et al. 2002). Supply chain coordination also involves cooperation between firms sharing important information with each other in the process of developing, producing and distributing goods and services to end marketplaces. Coordination can also be defined as structuring the efforts of a couple or more of supply chain drivers for the outcome of achieving effectiveness and be aware of each other’s tasks while working independently to achieve their actual set of goals (Ning et al. 2008). However, lack of coordination occurs in the supply chain, when each stage has incomplete information about the flow of products, information, and funds. Such causes will reduce the supply chain performance as a whole. Thus, supply chain coordination becomes vital to achieve the all level consensus, in which different members along a supply chain can respond to market requirements in proper ways (Chopra and Meindl 2004; Ninget et al. 2008).

Most of the previous researches in Ethiopia did not stress on the supply chain coordination. But this study puts its emphasis on this matter. Putting its focus on the dairy production portion of the agriculture in the country, suggests new ways approaching the sector, which will bring about enormous change both in the outlook and practice. The study also attempts to contribute in filling the gap of the studies made on this matter and proposed different mechanisms that could be used in coordination among milk cooperative unions, processors, and retail markets.

## Background of the study

**Fig. 1:**
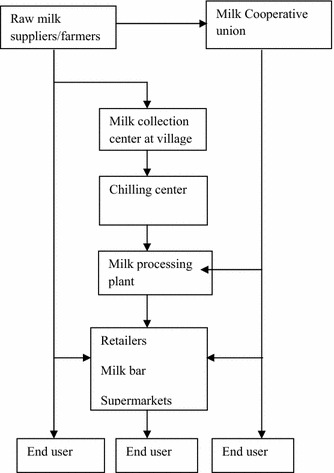
Dairy supply chain process in Ethiopia

The point of departure of this study emanates from the fact that the concept as well as the implementation of well-coordinated supply chain management is not developed in Ethiopia. Although it applies industry and agriculture led economies, the need of institutionalized supply chain coordination function is indispensable for Ethiopia as it attempts to transform its economy from agriculture to industry. This effort, among other things, calls for building self-capacity for managing, processing and supplying home grown agricultural items (Fig. 1).

Even though there are a number of dairy farms in Ethiopia, each produces to accommodate its own raw milk demand and they are unable to satisfy the majority of the consuming population through providing processed milk and dairy products (MoARD 2007; SNV 2013). One might ask how a country with such great wealth of livestock may have people who are unable to feed themselves well. Among other things, poor ways of farming due to lack of coordination between supply chain partners and not being able to find access to market, are the main constraints for the process of effective and efficient dairy production in Ethiopia. With this regard, the gap between the demand and the supply of available processed milk makes the researcher to investigate the major determinants of supply chain coordination in milk and dairy processing industries. Here, the question is how to achieve the strategic fit in the supply chain so that the tasks of each supply chain stage can be completed in a manner consistent with a mutual goal. The reason is that firms’ supply chain profitability depends on how well all supply chain members work together.

## Objective of the study

The study has been undertaken with the following objectives:

- To identify the key factors affecting the existence of supply chain coordination between suppliers, milk processing plants, and retailers.
- To examine the relationship between supply chain constructs and measured variables.
- To develop a model and suggest strategies to enhance coordination within the sphere of milk and dairy sectors.

## Literature review

In trying to elaborate the attributes of coordination, we can say that coordination in a supply chain involves putting the existing interdependencies in order (Li et al. 2002; Simatupang et al. 2008). Supply chain coordination involves cooperation between firms sharing important information with each other in the process of developing, producing and distributing goods and services to end marketplaces. Coordination of different business activities among units becomes vital as organizations pay much attention to their core activities. Thus, their fruitfulness constantly relies on their capacity to coordinate their internal and external activities in the value chain outside their own boundaries (Soroor et al. 2009).

The need for coordination is evident in supply chains, as companies forming a supply chain are dependent on the performance of other organizations. Supply chain coordination is achieved when a decision-maker, acting rationally, makes decisions that are efficient for the supply chain as a whole (Gupta and Weerawat 2006). Given the nature of the interdependence between units, coordination is a necessary prerequisite to integrate their operations to achieve the mutual goal of the supply chain as a whole as well as those of its units (Simatupang and Sridharan 2002).

### Coordination mechanisms

There is growing interest from industry and academic disciplines regarding coordination in supply chains, particularly addressing the potential coordination mechanisms available to eliminate sub-optimization within supply chains. However, there is a disconnect between what is known in academic research about coordination mechanisms and what mechanisms practitioners apply and consider useful (Fugate et al. 2010). The supply chain members are dependent on each other for resources and information, and this dependency has been increasing in recent times due to outsourcing, globalization and rapid innovations in information technologies. This increase in dependency brings some extent of risk and uncertainty too along with benefits. To meet these challenges, supply chain members must work towards a unified system and coordinate with each other. Here supply chain partners need to identify the coordination mechanisms which help in addressing the uncertainty in supply chain and achieving supply chain coordination (Arshinder and Deshmukh 2008) (Table 1).

**Table 1:** Factors affecting supply chain coordination

Major factors	Researchers
Harmonization of conflict	Achrol and Gregory (1999), Carson Stephen et al. (1999), Gundlach et al. (1995), Houston and Johnson (2000), Jap Sandy and Ganesan (2000), Lusch and Brown (1996), Maloni and Benton (2000), Poppo and Zenger (2002), Johnson Jean (1999)
Alliance	James and Ronchi (2002), Iyer (1998), Tsay and Agrawal (2000), Cao et al. (2008)
Quantity flexibility	Lariviere (1999), Fugate et al. (2010), Eppen and Iyer (1997), Tsay (1999), Bassok and Anupindi (1997)
Behavioral obstacle	Chopra and Meindl (2004), Himanshu et al. (2012)
Decentralized decision	Zimmer (2002), Sahin and Robinson (2002), Towill et al. (1992)
Information sharing	Chen (1998), Gavirneni et al. (1999), Dejonck et al. (2004), Ferguson and Ketzenberg (2006), Bourland et al. (1996), Achrol and Gregory (1999), Carson Stephen et al. (1999)
Mutual benefit	Gundlach et al. (1995), Houston and Johnson (2000), Jap Sandy and Ganesan (2000), Lusch and Brown (1996), Maloni and Benton (2000), Poppo and Zenger (2002), Johnson Jean (1999)
Collaboration relationships	McLaren et al. (2002), Walter et al. (2001)
Incentives	Sahin and Robinson (2002), Simatupang and Sridharan (2002), Himanshu et al. (2012)
Quantity discount	Chopra and Meindl (2004), Himanshu et al. (2012), Haghighat (2008), Iyer (1998), Tsay and Agrawal (2000), Cao et al. (2008)
Organizational interdependencies	Xu and Beamon (2006)
Price fluctuation	Chopra and Meindl (2004), Himanshu et al. (2012)
Cross functional team	Lambert and Cooper (2000), Whang (1995)
New product development	Hilletofth and Eriksson (2011), Cheng and Shiu (2008), Cooper et al. (2004), Droge et al. (2008), Hamm and Symonds (2006), Anderson and Narus (1990), Cachon and Lariviere (2005), Bianchi and Saleh (2010)
Trust	Arshinder and Deshmukh (2007), Singh (2011)

The studies carried out by Monczka et al. (1998), Young-Ybarra and Wiersema (1999), and Murali et al. (2011) have brought out that trust is the only force which binds all the parties to reap mutual benefits. However, Zimmer (2002), attempted to find a coordination mechanism which may help to improve decentralized decision-making. The study revealed that in a situation where decentralized decision-making existed, coordination was essentially required for lowering the total costs of supply chain in comparison to a centralized system. The performance level of the decentralized system was the same as that of a centralized system when a coordination mechanism for information sharing and incentives was employed in the study. The study came out with the conclusion that the correct and effective use of coordination mechanisms leads to optimal supply chain performance in supply chains with decentralized decision-making.

Himanshu et al. (2012) in their research study, considered the customer to be an integral part of the supply chain. Any supply chain is required to satisfy customer needs while generating the profit for itself. Supply chain activities start with an order from the customer and finish with a satisfied customer. Coordination is essential between the suppliers, processors and distributors for effective SCM. The elements such as inventory maintenance, replenishment and lead times are equally significant for fluctuation of orders and transportation costs.

*Trust* Trust can come when a company believes its business partner that will result in positive benefits for the maturation of both companies. When firms focused on a continuous relationship, the level of trust between both parties will be increased in a favorable way (Cullen et al. 2000). Thus, trust and information sharing is required for the smooth flow of information and enhances supply chain coordination as a whole (Cachon and Lariviere 2005; Bianchi and Saleh 2010; Arshinder and Deshmukh 2007; Singh 2011). Many researchers also consider trust as the most important component in supply chain coordination and alliance relationship (Chopra and Meindl 2004).

Hilletofth and Eriksson (2011) undertook their study on coordinating new product development with supply chain management. The study emphasized on the need to produce innovative and value-adding products. The prompt delivery of these products in the market is equally important. The companies facing mature business environments may face difficulties due to greater emphasis on other value-creation processes or on the value delivery processes. Therefore, new product development activities need to be coordinated with firms’ supply chain activities on a strategic level (Van Hoek and Chapman 2007). Consumer-desired products need to be produced by the firms in order to be competitive in the market. These products also need to be brought to the marketplace efficiently and effectively in a convenient way (Kotler et al. 2009).

Supply chain coordination is also required when starting a new product at the earliest stage. The value advantage, price, technical progress, and innovativeness are common product features that bears upon the success of the product (Cheng and Shiu 2008; Cooper et al. 2004; Droge et al. 2008; Hamm and Symonds 2006; Henard and Szymanski 2001; Kotler et al. 2009; Van Kleef et al. 2005). And besides, it is the consumer-perceived value, in contrast to the consumer-perceived cost, which decides what product characteristics are vital for success.

*Quantity flexibility* is one of the most widely discussed forms of non-price coordination. Quantity flexibilities allow the buyer to get a different quantity than the earlier estimate (Lariviere^1999^; Fugate et al. 2010) and this can be provided in various ways, such as minimum purchase quantity agreement (Bassok and Anupindi 1997), backup agreements that allow a customer to purchase higher quantities than initial amounts they ordered (Eppen and Iyer 1997) Quantity flexibility also considered as a major form of supply chain agreement (Tsay 1999). Sharafali and Co (2000) also suggested different types of coordination systems, for instance, price fluctuation and quantity discounts. Apart from it, the determinants such as flexibility, mutual benefit, harmonization of conflict, and information sharing are also found to be crucial in coordinating supply chains (Achrol and Gregory 1999; Carson Stephen et al. 1999; Houston and Johnson 2000; Jap Sandy and Ganesan 2000; Maloni and Benton 2000; Poppo and Zenger 2002).

On the other hand, coordination in each stage of the supply can be effective during cross-functional integration. Hence, successful supply chain coordination requires cross-functional integration in various supply chain activities (Lambert and Cooper 2000).

*Information sharing* Coordination between different stages of supply is very important for the success of the global business optimization, and it is only achieved if supply chain members share their information unambiguously. The importance of information sharing within a supply chain has been extensively analyzed by different scholars. The studies carried out by them have used simulation to assess the value of information sharing in the supply chain coordination (Towill et al. 1992; Bourland et al. 1996; Chen 1998; Gavirneni et al. 1999; Dejonck et al. 2004; Ferguson and Ketzenberg 2006). Simatupang and Sridharan (2002) also stated different forms of supply chain coordination such as, information sharing, and incentive alignment. These coordination methods are imperative to assist supply chain members and enhance sustainable supply chain profitability.

Sahin and Robinson (2002) identified centralized decision-making and decentralized decision-making for better-utilizing supply chain coordination. Equally opposed to centralized decision, decentralized decision-making is the best direction for better supply chain coordination as well as for prompt customer order fulfillment. Coordination mechanisms can also be classified into price and non-price coordination. Price coordination includes quantity discount and price fluctuation whereas; non-price coordination includes quantity flexibility, alliance and harmonization (Iyer 1998; Tsay and Agrawal 2000).

Haghighat (2008) suggested quantity discount as a method for coordinating the order quantity between a retailer and supplier. But the motivation for giving quantity discounts might be either based on price discrimination or order quantities. On the other hand, the alliance is also a way of supply chain coordination in which both buyers and sellers can be benefited by providing value to each other. According to Rice and Ronchi (2002), if there is alliance in the supply chain, business partners can share some mutual interest, exchange value through buyer–seller activities, and also perform some coordination mechanisms.

Xu and Beamon (2006) supply chain coordination is a strategic weapon to the problems that occurs from inter-organizational dependencies within the chain. Whang (1995) also carried out research on the taxonomy of coordination and he suggested cross-functional and inter-organizational team as different level of coordination mechanisms. Collaboration is a recent trend in supply chain management that focuses on joint planning, coordination, and process integration between suppliers, customers and other partners in a supply chain (McLaren et al. 2002). Walter et al. (2001) observed that the high performing collaboration relationship required not only a focus on the direct value creating or buyer–supplier function, but also an equal emphasis on the indirect relationship building and sustaining function. The study conducted by Christopher (1999), also shows that companies are moving towards a collaborative relationship in an attempt to make the supply chains more competitive.

## Research methodology

### Research design and scale development

The methodology of the paper is quantitative in nature. A survey research design was used to collect data for the scale development. Items were developed based on extensive literature review and consulting with supply chain professionals. The items were also measured by conducting a pilot test on some other milk industries and the researchers have also discussed with supply chain practitioners and with those people who have engaged themselves in milk processing.

### Study area and population

The survey had been conducted mainly on the supply of milk to the inhabitants of Addis Ababa from the nearby rural districts. The study focuses on suppliers, local milk processing industries, and retailers. Accordingly, the study covers the north part of the capital, South East, and South West where potential milk supply comes from especially from local farmers association such as, Selale cooperative union, Ada‘a Liben cooperative union and Sebata area. On the other hand, the major dairy processors such as lame dairy (sholla), mama dairy (sebata agro-industry), and family dairy are included in the study. In addition, around 15 retail markets/milk bars in Addis Ababa town are also parts of study. For methodological reasons, hence in line with the objectives, the general population of this study includes all actors in the milk industry along the chain of market.

### Sampling and data collection

The sample was drawn from suppliers, processors and retailers in Bishoftu, Selale, and Addis Ababa cities. The data collection instrument used was a questionnaire which was administered to the total sample size of 375 respondents. Of the 375 distributed items, 15 were returned due to an unwillingness of respondents. From the sample size of 360, 342 were received, resulting in a response rate of 95 %. A total of eight questionnaires were discarded because of incomplete data. Therefore, only 330 respondents were considered as valid and the result represented an accurate response rate of 91.6 %. Out of 330 respondents, the study included 225 (68 %) milk suppliers, 75 (23 %) retailers and 30 (9 %) employees from three major milk processing plants (Shola milk, Mama & Family dairy). A seven-point Likert scale with end points of “Strongly disagree” and “Strongly agree” was applied to measure the items. The sample was drown using stratified sampling techniques.

### Reliability

The Cronbach’s alpha was conducted to evaluate the reliability of each scale. Alpha values over 0.7 indicate that all scales can be regarded as reliable (Hair et al. 2010). As can be seen from Table 2, Cronbach’s alpha value of coordination is 0.807 and the scale alpha values of the four factors were above the cutoff value, ranging from 0.963 to 0.979. These results imply that the theoretical constructs are good indicators of the model fit. Thus, we can state that the instrument is acceptable and used to measure 15 coordination variables.

**Table 2:** Factor analysis result for key coordination indicators

Variables	Factors			
	Non price coordination	Relationship	Price coordination	Product devp’t decision
Harmonization conflict	0.968			
Behavioral obstacle	0.964			
Quantity flexibility	0.961			
Alliance	0.946			
Decentralized decision	0.899			
Information Sharing		0.948		
Mutual benefit		0.938		
Incentives		0.935		
Collaboration		0.903		
Quantity discount			0.935	
Organizational interdependence			0.924	
Price fluctuation			0.908	
Cross functional team				0.954
New product development				0.949
Trust				0.877
Cronbach’s alpha	0.979	0.975	0.963	0.967
Egin value	4.642	3.728	2.796	1.937
Percentage variance	92.472	93.19	93.179	96.828

KMO (Kaiser–Meyer–Olkin) measure of sampling adequacy = 0.819

Bartlett’s test of sphericity (χ^2^ = 7829.503, Df = 105, Sig = 0.00), mean = 79.91

### Scale refinement

For each of the item scales, factor analysis was applied to reduce the total number of items in manageable factor. A principal component analysis is applied to extract factors with an Eigenvalue greater than 1. Varimax rotation is employed to facilitate interpretation of the factor matrix. Kaiser–Meyer–Olkin (KMO) measure of sampling adequacy also examined to validate factor analysis. The KMO value was estimated around 0.819 which indicates sampling adequacy. The factor loading indicates four distinct constructs: non-price coordination (F1), relationship (F2), price coordination (F3), product development decision (F4) (Table 3).

**Table 3:** Mean, SD, corrected item-to-total correlation and communality for key coordination indicators

Variables	Mean	Std. deviation	Corrected item-total correlation	Alpha if item deleted	Communality	
					Initial	Extracted
Harmonization of conflict	5.4	0.601	0.965	0.776	1.00	0.955
Quantity flexibility	5.41	0.624	0.996	0.777	1.00	0.945
Alliance	5.38	0.608	0.915	0.778	1.00	0.912
Behavioral obstacle	5.41	0.623	0.975	0.776	1.00	0.949
Decentralized decision	5.37	0.681	0.873	0.782	1.00	0.828
Information sharing	5.28	0.69	0.97	0.789	1.00	0.97
Mutual benefit	5.26	0.709	0.938	0.791	1.00	0.933
Collaboration	5.24	0.724	0.884	0.795	1.00	0.87
Incentives	5.27	0.696	0.959	0.788	1.00	0.958
Quantity discount	5.25	0.503	0.947	0.807	1.00	0.955
Organizational interdependence	5.25	0.513	0.934	0.808	1.00	0.944
Price fluctuation	5.27	0.502	0.885	0.809	1.00	0.898
Cross functional team	5.37	0.762	0.937	0.818	1.00	0.969
New product development	5.39	0.749	0.937	0.816	1.00	0.968
Trust	5.35	0.632	0.856	0.781	1.00	0.784

Scale statistics: mean = 79.91, variance, 25.183, Std. deviation = 5.018, number of variables = 15, number of cases = 330, Cronbach’s alpha = 0.807

## Results

As we have seen in the above table, item-to-total correlation range 0.996–0.856 and the commonality ranges above 0.5. The mean score value is 79.91 with 25.183 variance and 5.018 Std Deviation. And also, the total scale reliability alpha is 0.808, which is greater than 0.6 and confirmed the reliability of the questionnaire.

### Factor analysis result

#### Non price coordination (F1)

This factor covers five key coordination indicators (KCI). These are Harmonization of Conflict, Alliance, Behavioral Obstacles, and Quantity Flexibility and Decentralized Decision. The factor loading ranges from 0.968 to 0.899 and the Cronbach’s alpha value is 0.978. Item-to-total correlation ranges from 0.873 to 0.975. Here, 92.472 % of the division is explained and it covers 4.642 of the Eigenvalues.

#### Relationship (F2)

The relationship factor covers four KCI. These are Information Sharing, Mutual Benefit, Incentives, Collaborative Relationship, and Quantity Discount. The factor loading ranges from 0.948 to 0.903 and the Cronbach’s alpha value is 0.975. Item-to-total correlation ranges from 0.970 to 0.884. Here, 93.19 % of the variance is explained and it covers 3.728 of the Eigen values.

#### Price coordination (F3)

Three measured variables are identified in price coordination factor. These are quantity discount, organizational interdependencies, and price fluctuation. The factor loading ranges from 0.947 to 0.885 and the Cronbach’s alpha value is 0.963. Item-to-total correlation ranges from 0.935 to 0.908. Here, 93.179 % of the variance is explained and it covers 2.796 of the Eigenvalues.

#### Product development decision (F4)

This factor covers three KCI. These are cross functional team, new product development, and trust. The factor loading ranges from 0.857 to 0.669 and the Cronbach’s alpha value is 0.737. Corrected item-to-total correlation ranges from 0.954 to 0.877. Here, 96.828 % of the variance is explained and it covers 1.937 of the Eigen values.

The correlations between constructs and indicators (Table 4) show acceptable discriminant validity, as correlations between constructs (non price coordination, relationship, price coordination and product development decision) and their defining indicators (summated 1, 2, 3, and 4) are highly significant while correlations between indicators and the remaining constructs are low and insignificant.

**Table 4:** Correlation

Correlation between constructs and indicators								
Non price coordination	1.00							
Information and r/ship	0.504	1.00						
Price coordination	0.498	0.389	1.00					
Product development	−0.004	0.188	−0.08	1.00				
Summated 1	0.913*	0.233	0.279	−0.018	1.00			
Summated 2	0.256	0.926*	0.145	0.102	−0.000	1.00		
Summated 3	0.251	0.228	0.922*	−0.036	−0.009	0.000	1.00	
Summated 4	−0.004	0.118	−0.069	0.983*	0.000	0.000	0.000	1.00

* Correlation is significant at 0.01 level (two-tailed)

Italic values indicate the highest correlation among constructs and summated scales

### Confirmatory factor analysis result

**Fig. 2:**
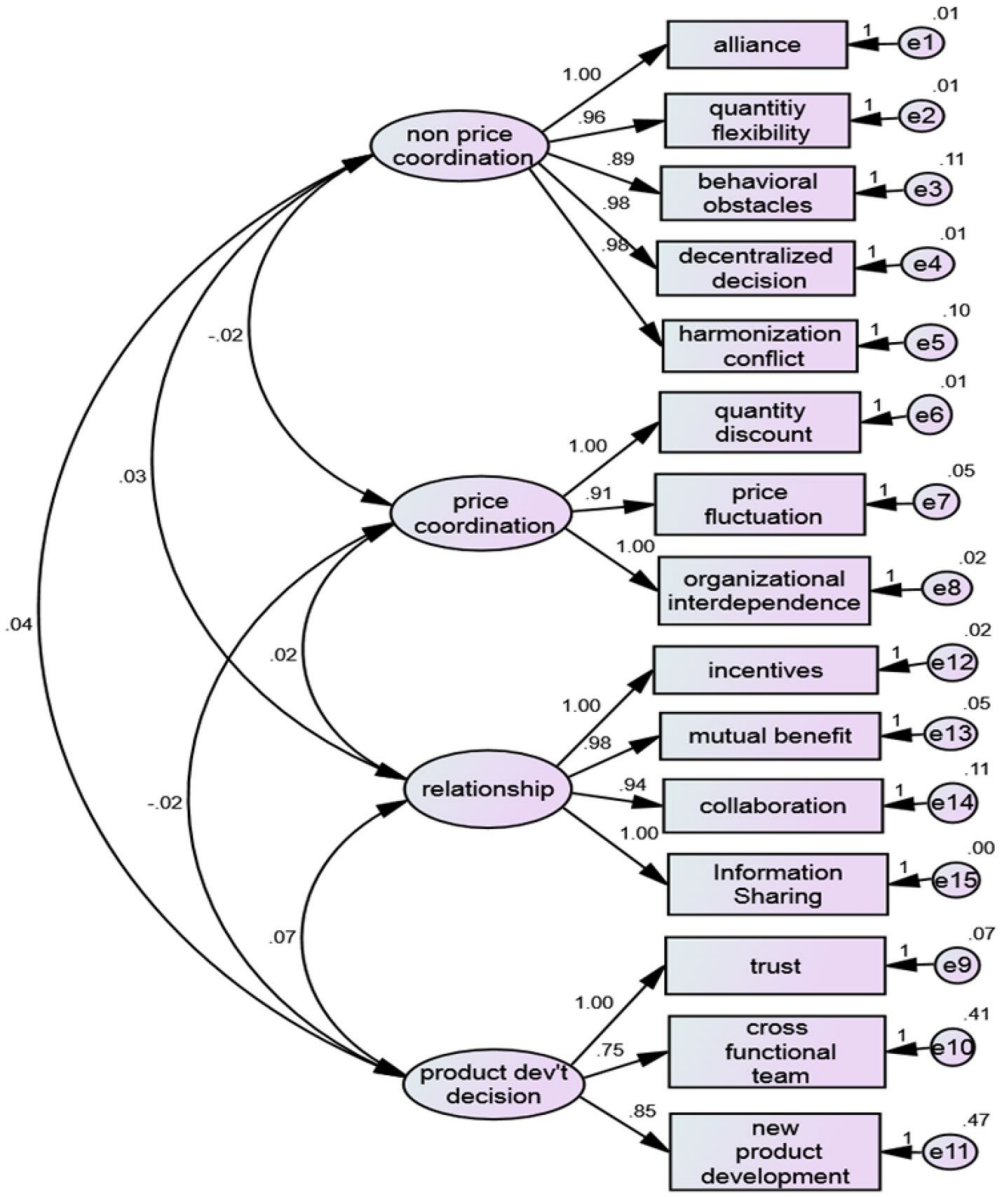
Confirmatory model: unstandardized result

**Fig. 3:**
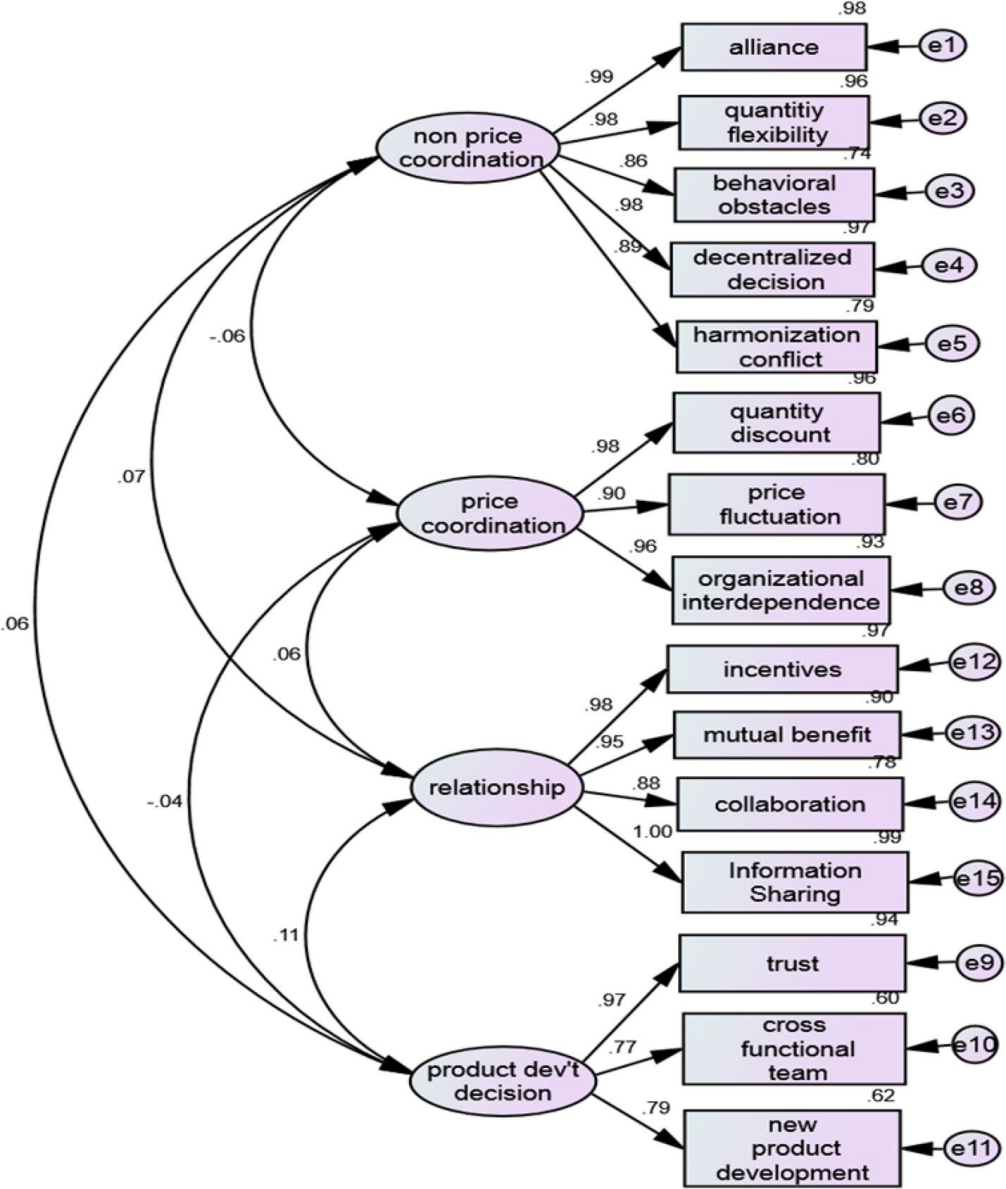
Confirmatory model: standardized result

Confirmatory factor analysis is appropriate to analyze how well the measured variables/items clearly represent the latent constructs (Hair et al. 2010). In such case, the confirmatory model loadings are illustrated with Standardized and Unstandardized results (Figs. 2, 3). This confirmatory model was estimated by maximum likelihood (ML) and the model fit results are discussed. The overall fit of the models was examined by various indices and the results of the Standardized and Unstandardized model were *X*^2^ = 161. 809; *Df*, 84; *P* value = 0. 000.

*Root mean square residual* Lower RMR represents a better fit, but higher value indicates worse fit (Hair et al. 2010). The RMR estimate of the present study was 0.008, meaning a reasonable fit.

*Root mean square error of approximation* It is useful to adjust the complexity of models and to manage the tendency of the chi-square goodness of fit (Kline 2005; Hair et al. 2010). In this field, the RMSEA estimate is 0.047. Goodness of fit index (GFI): the acceptable range of GFI value is between 0 and 1 and the higher value indicates the better fit (Kline 2005). The GFI estimate for the current study is 0.943, which represent a good indicator of model fit.

*Normal fit index* ranges between 0 and 1 and a model approaching 1 represent the perfect fit (Hair et al. 2010). In this study, the NFI value is 0.977, which is a significant and a good indicator of model fit.

*Comparative fit index* It is also widely used indices which help to compare the proposed model with baseline model and model values above 0.90 represents a good indicator of model fit (Kline 2005). In the present study, CFI value represents 0.989; this is another indicator of model fit.

### Standardized confirmatory model

As indicated on the standardized confirmatory model (Fig. 3), the four major constructs are non-price coordination, relationship, price coordination and product development decision. These factors are identified in the following section briefly;

*Non price coordination* This construct consists of 5 measured variables such as alliance (0.99), decentralized decision (0.98), harmonization conflict (0.89), quantity flexibility (0.98) and behavioral obstacles (0.86). All the loading points were calculated within the range 0.86–0.99. This reveals that alliance, decentralized decision, and quantity flexibility play a key part for the betterment of supply chain coordination among firms procurement, production and distribution systems. These determinants also help to coordinate raw milk producers, processors, and retailers through team-based approaches. Hence, in supply chain coordination, suppliers and processors must have a smooth relationship with distributors/retailers that compete not only in monetary value, but also in non-price coordination manner.

*Relationship* Here, the loading point of measured variables ranges from 0.88 to 1.00. In this case, information sharing (1.00), incentives (0.98) and mutual benefit (0.95) are the major determinants of relationship coordination. The existence of collaboration (0.88) among producers, processors and retailers will also helpful for better supply chain coordination. As we know that, nowadays, it is hard to do business independently in which there exist many competitors. Thus, well-organized supply chain coordination is more desirable for sustainable business profitable. In this regard, long term supply chain relationship is one of the keys to success.

*Price coordination* This section helps us to better understanding of financial flows within the supply chain stages through price coordination mechanism. As we have found out in the confirmatory model, the factor loading point of the quantity discount and organizational interdependencies were 0.98 and 0.96, respectively. In addition, price fluctuation represents 0.90, meaning that all measured variables have a significant contribution to price coordination. In the case of milk and dairy supply chain, the volume of milk supply can be affected by seasonality of demand, shortages of supply and some other environmental elements. It is too true that price fluctuation and quantity discounts are among the major factors that can adversely affect supply chain coordination as a whole. Thus, firms’ supply chain strategies should be supported by financial resource and this will create an economic link and organizational interdependence between suppliers and local milk processing industries.

*Product development decision* Here, three measured variables are explained under product development decision construct. As depicted in the model, the overall loading for each item ranged between 0.94 and 0.60 and the loading of trust was set at 0.94 and new product development as well as cross functional team explained about 0.62 and 0.60, respectively. New product development decision, trust and cross functional team activities need to be coordinated with supply chain management at a strategic level so that less competitiveness in the supply chain will be decreased.

The proposed research framework was tested using Confirmatory Factor Model. All indices were significant and greater than the threshold value, and then we can state that the results are in a good fit. Table 5 depicts the un-standardized and standardized result for each hypothesized path, along with statistical indices.

**Table 5:** Model fit summary

Indices	Fit indices for the measurement model		
	Unstandardized result	Standardized result	Recommended values
χ^2^	161.809	146.029	
Df	84	84	
χ^2^/df	1.92	1.73	<3.0
P-value	0.000	0.000	
RMR	0.008	0.008	<0.10
GFI	0.943	0.948	>0.90
AGFI	0.919	0.926	
NFI	0.977	0.978	
RFI	0.972	0.972	
IFI	0.989	0.99	
TLI	0.986	0.998	Values that approach to 1
CFI	0.989	0.99	>0.90
RMSEA	0.047	0.047	Between 0.03 and 0.08
AIC	233.809	218.029	<Saturated and independence models
AIC saturated model	240	240	<Saturated and independence models
AIC independent model	7139.849	6619.771	

## Discussions

This study significantly contributes to the supply chain literature through analyzing the determinants of supply chain coordination and its impact on sustainable business profitability. It is necessary that we consider the importance of coordination in all the dealings of supply chain management in order to guaranty an assured mutual gain. In regard to the findings of this study, the first most important group of Key Coordination Indicators (KCI) is categorized under *non*-*price coordination* metrics. This factor consists of five key coordination variables. The other nodal point is the *relationship* construct. Supply chain relationships also play a pivotal role to create integration between each of the supply chain stages. Chopra and Meindl (2004) also confirms that when such relationship is adopted in between firms, the level of trust between supply chain partners will be maximized in the desired way. Accordingly, the relationship construct covered four key coordination indicators, namely *Information Sharing*, *Collaboration*, *Mutual Benefit*, *and Incentive*.

The other aspect is that price coordination covers three key measured variables, namely *Organizational Interdependence*, *Price Fluctuation*, *and Quantity Discount*. Here, two measured variables relating to price coordination, such as, *Sales Promotion* and *Price stability* were deleted from the final instrument. Therefore, price coordination construct did not include sales promotion and price stability variables. But further research shall be extended to these variables by examining in a different perspective. In addition, the Product development decision presents the last nodal point for measuring SC coordination. *Trust*, *New Product Development*, and *Cross*-*functional Teams* were the major measured variables in product decision matrix. These outcomes can vitally be used in evaluating the major roles of milk processing industries and in identifying the gap in the problem area. The results can also be used as a strategic weapon to distinguish the main problem areas in which each and every change in betterment are required so that milk industries can easily implement their supply chain strategies in association with their business partners.

## Conclusion

In previous decades the main and crucial stages of the supply chain such as procurement, production and distribution seem to have been dominantly managed independently. But the accessibility of excess inventories, intense competition, and market globalization were forcing firms to enhance their supply chain capabilities that can promptly respond to consumer preferences (Thomas and Griffin 1996). To cope up and endure in a business environment where competition is high, firms should decrease the flow of interruption within upstream and downstream supply chain activities. This kind of endurance in such a business environment can only be achieved by means of organized supply chain coordination. Supply chain coordination practice attracts most firms, mainly those operating businesses independently. It is something that every firm needs for managing interdependent logistic activities in order to mitigate demand variability and unnecessary inventories. Giving consideration to these obvious reasons, this study was undertaken to identify the key determinants of coordination indicators in milk and dairy industries of Ethiopia. The study created 15 measured variables and offered a comprehensive model for examining supply chain coordination. Based on a scrutinized literature review, it conceptualizes supply chain coordination as a major construct such as non-price coordination, relationship, price coordination, and product development decision (Additional file 1). Thus, firms should realize that its individual profitability and competitiveness depends heavily on supply chain coordination with its business partners. Therefore, there is no way that organizations run effectively while doing their businesses without coordination. That is why we strongly recommend that firms should apply the above indicated coordination mechanisms in each of their business dealings.

### Limitations and direction for future research

We can see the limitations of this study in two aspects. First, even though the study is done with regard to the milk suppliers, processors and retailers, the end product users (customers) are not incorporated. It is obvious that customers play an inevitable role in the profitability as well as success of a certain company and also for sustainable coordinated business. Therefore, future studies should put into consideration that final customers need to be involved as a major input for their study. The second aspect is that the study is not done based on the role of supply chain coordination on organizational performance. Future studies should use structural methods to investigate the matter based on the current performance of the given organizations.

## Additional file

**Additional file 1.** Appendix: Coordination measurement scale.
