# Supplementary material for: Determinants of supply chain coordination of milk and dairy industries in Ethiopia: a case of Addis Ababa and its surroundings
Source: Springerplus. 2015 Sep 16;4:498. doi: 10.1186/s40064-015-1287-x (PMC4573177; doi:10.1186/s40064-015-1287-x)
Supplement: Supplementary file 1 — Additional file 1. Appendix: Coordination measurement scale. [file 40064_2015_1287_MOESM1_ESM.docx]

**Appendix**

**Coordination measurement scale**

**A seven point (7) Likert scale with end points of "strongly disagree" and "strongly agree"**

**1= Strongly Disagree, 2= Disagree, 3=Disagree Somewhat, 4= Undecided /Neutral, 5=Agree Somewhat, 6=Agree, 7= Strongly Agree**

| **Note: Please rate the importance and effect of each factors affecting supply chain coordination, with (√) in the appropriate column.** | **Response** | | | | | | |
| --- | --- | --- | --- | --- | --- | --- | --- |
|  | **1** | **2** | **3** | **4** | **5** | **6** | **7** |
| **Non Price Coordination** | | | | | | | |
| NPC1:**Quantity flexibility** contracts are must in supply chain coordination |  |  |  |  |  |  |  |
| NPC2:We feel that **harmonization of conflict** is must for effective supply chain coordination |  |  |  |  |  |  |  |
| NPC3:we perceived **alliances** within the stage of supply as a factor affecting milk supply chain coordination |  |  |  |  |  |  |  |
| NPC4:we considers **behavioral obstacles** as a factor affecting milk supply chain coordination |  |  |  |  |  |  |  |
| NPC5:we believe in **decentralized decision** for speed flow of process |  |  |  |  |  |  |  |
| **Price Coordination** | | | | | | | |
| PC1: we perceive that **organizational interdependencies** positively affects milk supply chain coordination |  |  |  |  |  |  |  |
| PC2: we offers **quantity discounts** to attract more users |  |  |  |  |  |  |  |
| PC3: we perceive that **Price fluctuations** affects milk SC coordination |  |  |  |  |  |  |  |
| **Relationship** | | | | | | | |
| R1: we have good **collaborative relationships** with our partners |  |  |  |  |  |  |  |
| R2:we use **incentives** for better supply chain coordination |  |  |  |  |  |  |  |
| R3:we focus on **mutual benefit** to maintain SC coordination |  |  |  |  |  |  |  |
| R4: We and our trading partners **share information** that helps establishment of business planning |  |  |  |  |  |  |  |
| **Product Development Decision** | | | | | | | |
| PDD1:Our key business partners are involved in **new product development** |  |  |  |  |  |  |  |
| PDD2:we use **cross-functional teams** strategies with our SC partners for continuous product improvement |  |  |  |  |  |  |  |
| PDD3: We offer products that are very **trustworthy** |  |  |  |  |  |  |  |
